# IL-10 Production in Macrophages Is Regulated by a TLR-Driven CREB-Mediated Mechanism That Is Linked to Genes Involved in Cell Metabolism

**DOI:** 10.4049/jimmunol.1500146

**Published:** 2015-06-26

**Authors:** David E. Sanin, Catriona T. Prendergast, Adrian P. Mountford

**Affiliations:** Department of Biology, Centre for Immunology and Infection, University of York, York YO10 5DD, United Kingdom

## Abstract

IL-10 is produced by macrophages in diverse immune settings and is critical in limiting immune-mediated pathology. In helminth infections, macrophages are an important source of IL-10; however, the molecular mechanism underpinning production of IL-10 by these cells is poorly characterized. In this study, bone marrow–derived macrophages exposed to excretory/secretory products released by *Schistosoma mansoni* cercariae rapidly produce IL-10 as a result of MyD88-mediated activation of MEK/ERK/RSK and p38. The phosphorylation of these kinases was triggered by TLR2 and TLR4 and converged on activation of the transcription factor CREB. Following phosphorylation, CREB is recruited to a novel regulatory element in the *Il10* promoter and is also responsible for regulating a network of genes involved in metabolic processes, such as glycolysis, the tricarboxylic acid cycle, and oxidative phosphorylation. Moreover, skin-resident tissue macrophages, which encounter *S. mansoni* excretory/secretory products during infection, are the first monocytes to produce IL-10 in vivo early postinfection with *S. mansoni* cercariae. The early and rapid release of IL-10 by these cells has the potential to condition the dermal microenvironment encountered by immune cells recruited to this infection site, and we propose a mechanism by which CREB regulates the production of IL-10 by macrophages in the skin, but also has a major effect on their metabolic state.

## Introduction

Interleukin-10 is known to be critical in maintaining the balance between a strong prophylactic immune response and limiting immune-mediated pathology during many diseases caused by parasitic protozoa and helminths, as reviewed recently (1). The importance of IL-10 has been demonstrated particularly during human *Schistosoma sp.* infection (2–4) and in the murine chronic model of this disease (5–8). However, until only recently, the role of this cytokine had not been investigated during the early stages of schistosome infection, as the host’s skin is exposed to infective cercariae. The production of IL-10 increases substantially in the skin site of infection, especially after repeated exposure to cercariae (9), and is responsible for the induction of CD4 T cell hyporesponsiveness in the skin draining lymph nodes (10) and prevention of excessive tissue damage/inflammation in the skin (11). Although CD4^+^ T cells (often CD25^+^) are the primary cellular source of IL-10 during the chronic phase of *Schistosoma mansoni* infection (1, 12), shortly after exposure of the skin to *S. mansoni* cercariae, both tissue macrophages and CD4^+^ T cells were reported to produce IL-10 (11). However, the molecular mechanism underpinning production of IL-10 by macrophages has not been fully characterized.

Macrophages and dendritic cells (DCs) produce IL-10 in response to TLR and C-type lectin receptor ligands (13–15). The mechanism that controls IL-10 production in these cells in response to defined stimuli (e.g., LPS and zymosan) is thought to involve MAPKs, such as ERK, p38, mitogen and stress-activated protein kinases (14, 16), and transcription factors, like CREB, NF-κB p50 homodimers, and C/EBPβ (13, 16–19). In addition to stimulation of TLR and C-type lectin receptor on the cell surface, macrophages are phagocytic and constantly sample their environment by actively internalizing foreign macromolecules by endocytosis. Endocytosis is tightly regulated as it is energy costly (20), and it modulates downstream signaling pathways (21, 22). However, the role of Ag uptake is only partly understood in the context of TLR signaling, and the impact of endocytosis on the production of IL-10 has not been studied.

During *S. mansoni* percutaneous infection, the earliest sources of Ag to interact with innate immune cells in the skin are cercarial excretory/secretory (E/S) products (0–3 h released products [0–3hRP]), which are released by cercariae as they penetrate the host (23, 24). These E/S products contain >70 different proteins (25, 26), some of which are glycosylated (27), but only a few have defined roles in assisting extracellular matrix remodeling (24, 28, 29), or modulating innate immune cells (30–32). Indeed, macrophages, as well as DCs, are among the earliest cells in the skin to take up cercarial E/S products (23). These E/S products induce the production of various cytokines by macrophages in vitro (32–34), for which MyD88 and TLR4 are important (34), but their ability to specifically induce IL-10 is not known.

In this study, the molecular mechanism underpinning production of IL-10 by bone marrow–derived macrophages (BMMs) exposed to E/S products released by *S. mansoni* cercariae was investigated. We demonstrate that rapid production of IL-10 results from MyD88-mediated activation of two branches of the MAPK signaling pathway, MEK/ERK/RSK and p38, following ligation of TLR2 and TLR4. Moreover, these kinases converge upon activation of the transcription factor CREB, which is critical for production of IL-10. We show that CREB is recruited to a novel regulatory element in the *Il10* promoter as a consequence of macrophage stimulation with 0–3hRP and that it regulates a network of genes involved in metabolic processes. Finally, we investigated the production of IL-10 in vivo by monocytes in the skin early postinfection with *S. mansoni* cercariae and suggest a possible mechanism by which macrophages in the skin produce IL-10, which in turn is involved in the regulation of their metabolic state.

## Materials and Methods

### Animals

Wild-type C57BL/6 (WT), IL-10^−/−^ (IL-10–deficient) (35), TLR2^−/−^ (TLR2-deficient) (36), as well as transgenic IL-10 reporter knockin (*tiger*) (IL-10^+/GFP^) (37) mice were bred and housed at the University of York (York, U.K.). Bone marrow from TLR4^−/−^ or MyD88^−/−^ strains (38, 39) was obtained from mice housed at the University of Edinburgh (Edinburgh, U.K.), whereas bone marrow cells deficient in tumor progression locus 2 (TPL2; Tpl2^−/−^) (40) were obtained from animals housed at the National Institute for Medical Research (London, U.K.). All transgenic strains were on a B6 genetic background. Female mice aged between 6 and 10 wk were used for all experiments carried out in accordance with the U.K. Animal’s Scientific Procedures Act 1986 and with approval of the University of York Ethics Committee.

### Parasites and parasite-derived material

Infective *S. mansoni* cercariae (Puerto Rican strain) were obtained from *Biomphalaria glabrata* snails exposed to incandescent light for 2 h. Cercariae were collected, washed with chilled filter-sterilized water, and used to collect cercarial E/S products as described previously (32, 34, 41). Mechanically transformed cercariae were cultured for 3 h and the resulting culture supernatant containing cercarial E/S products concentrated using filter spin columns with an m.w. cutoff of 3 kDa (GE Life Sciences, Pittsburgh, PA). This concentrated supernatant was termed 0–3hRP (34, 41) and its protein content determined by BCA protein assay (Thermo Scientific, Waltham, MA). In selected experiments, 0–3hRP was conjugated to Alexa Fluor 633 (Life Technologies, Paisley, U.K.) (0–3hRP^AF633^) by incubating together for 3 h (1 μg of dye per 100 μg 0–3hRP) and then removing excess dye with a filter spin column.

### In vitro culture and stimulation of BMMs

Macrophages were derived from the bone marrow using a well-established experimental system; methodological details defined as being critical during cell culture (42) are given below. Aliquots of bone marrow cells (5 × 10^6^) were cultured for 7 d (37°C 5% CO_2_) in DMEM (Life Technologies) containing 10% heat-inactivated FCS (Biosera, Uckfield, U.K.), 2 mM L-glutamine solution, 50 U/ml penicillin, 50 μg/ml streptomycin (all Life Technologies), and 50 μM 2-ME (Sigma-Aldrich, Gillingham, U.K.). Cell cultures were supplemented with CSF-1 obtained from culture supernatants of L929 murine fibroblast cell line. After 7 d, adherent cells were harvested and subsequently used as BMMs.

BMMs were cultured in complete DMEM (1 × 10^5^ cells/ml) and stimulated for 10–1000 min with 50 μg/ml 0–3hRP [low endotoxin 0.0015 ± 0.0003 μg/ml (34)], 0–3hRP^AF633^, or medium control (Media), all supplemented with 2 μg/ml polymixin B (Sigma-Aldrich) (34, 41). Additionally, BMMs were stimulated with 1 ng/ml LPS (Sigma-Aldrich), 25 μg/ml polyinosinic-polycytidylic acid (Sigma-Aldrich), or 5 μg/ml Pam3CSK4 (Invivogen, San Diego, CA). In selected experiments, BMMs were pretreated for 2 h with MEK inhibitor U0126 (3–30 μmol), p38 inhibitor SB203580 (0.3–3 μmol), PI3K family inhibitor LY294002 (25 μmol) (all from Cell Signaling Technology, Leiden, the Netherlands), or p110-δ inhibitor IC87114 (5 μg/ml) kindly donated by Dr. Klaus Okkenhaug (University of Cambridge, Cambridge, U.K.). Glucose in culture supernatants was measured with a Glucose (GO) assay kit (Sigma-Aldrich), whereas lactate was estimated using a Lactate colorimetric assay kit (BioVision, Mountain View CA). Hexokinase activity (in units per milliliter) of stimulated cells was measured using a Hexokinase colorimetric assay (BioVision).

### Infection protocol and recovery of dermal exudate cells

Mice were percutaneously exposed via each pinna to a single dose of 150 *S. mansoni* cercariae and pinnae harvested 1, 2, or 4 d postinfection. Pinnae from naive and infected mice were removed and split along the central cartilage into two halves to obtain the population of dermal exudate cells (DEC), as described previously (9, 43).

### Flow cytometry

Cells were blocked with 1 μg anti-CD16/32 mAb (eBioscience, Hatfield, U.K.) in goat serum (Sigma-Aldrich) and then labeled with LIVE/DEAD Fixable Aqua Dead Cell Stain (Life Technologies), plus the following mAbs conjugated to various fluorescent labels: anti–MHC class II (MHC-II; IA-IE) (clone M5/114), anti-CD11b (clone M1/70), and anti-F4/80 (clone BM8) (all from eBioscience). For intracellular staining of phosphorylated proteins, cells were washed, fixed with 2% paraformaldehyde, and incubated in 1× permeabilization buffer (eBioscience) with polyclonal Abs against ERK1/2, p-ERK1/2, p38, p-p38, or mAbs against RSK (clone 32D7), p-RSK (clone D5D8), CREB (clone 48H2), p-CREB (clone 87G3), p-p65 (93H1), and p-p65 (18E6) (all from Cell Signaling Technology). Finally, BMMs were incubated in 1× permeabilization buffer with Alexa Fluor 488–conjugated goat anti-rabbit Ab (Life Technologies). All flow cytometry was acquired using the Cyan ADP analyzer (DakoCytomation, Stockport, U.K.), or BD LSR Fortessa analyzer (BD Biosciences, Oxford, U.K.) and data analyzed using FlowJo software v7.6.5 (Tree Star, Ashland, OR).

### Detection of intracellular IL-10 in DEC

WT and IL-10^+/GFP^ mice were infected and pinnae harvested as described above. Split pinnae were incubated with complete RPMI 1640 for 12 h prior to the addition of 1× Brefeldin A (eBioscience) for a further 8 h. DEC were then prepared for flow cytometric analysis as described above.

### Cytokine analysis by ELISA

Culture supernatants were collected from BMM cell cultures, and IL-10, IL-12p40, and IL-12p70 were quantified by DuoSet ELISA (R&D Systems, Abingdon, U.K.).

### Confocal microscopy and immunofluorescence

BMMs were allowed to adhere to glass cover slips for 2 h in 24-well plates (1 × 10^6^ cells/well) prior to stimulation with 0–3hRP^AF633^ (50 μg/ml) for 60 min. Cells were washed, fixed on to cover slips for 20 min with 4% paraformaldehyde in PBS at room temperature, and placed in 0.05% saponin 0.2% BSA (staining buffer) for 30 min at room temperature before being incubated for 1 h with polyclonal rabbit Ab against early endosome Ag (EEA)-1 (Abcam; 1:200). Cover slips were washed three times and then probed for 1 h with goat anti-rabbit Alexa Fluor 488 (Life Technologies) (1:1000), washed as before, and counterstained with DAPI (2 μg/ml) (Sigma-Aldrich). Cover slips were mounted onto glass slides using Prolong Gold (Life Technologies). Images were acquired using a Zeiss 710 inverse confocal microscope (Carl Zeiss, Cambridge, U.K.) and all analyzed using identical acquisition settings in Zeiss ZEN software (Carl Zeiss). Image handling (including contrast adjustment) was conducted using ImageJ (National Institutes of Health).

### RNA extraction and gene expression analysis by PCR

RNA from stimulated BMMs (2 × 10^6^) was obtained using the High Pure RNA isolation kit (Roche, Burgess Hill, U.K.) and quantified using a Nanodrop (Thermo Scientific). Purified RNA was reverse transcribed into cDNA using SuperScript (Life Technologies) and the resulting cDNA analyzed for gene expression via quantitative real-time PCR using Fast SYBR Green Master Mix (Life Technologies) and primers (0.8 μmol) for *Il10* (forward [Fw]: 5′-GGTCTTGGGAAGAGAAACCAG-3′ and reverse [Rv]: 5′-GCCACAGTTTTCAGGGATGA-3′) and *Il12b* (Fw: 5′-ATCAAGAGCAGTAGCAGTTC-3′ and Rv: 5′-TACTTCTCATAGTCCCTTTG-3′). For early growth response protein 1 (*Egr1*), FBJ osteosarcoma oncogene (*Fos*), and NF-κB 2 (*Nfkb2*), primers were designed and validated directly by the manufacturer (Qiagen, Venlo, the Netherlands). Changes in gene expression after quantitative real-time PCR using a StepOnePlus PCR system (Life Technologies) were calculated using the δ-δ threshold cycle method (44) with *Gapdh* (Fw: 5′-CCATGTTTGTGATGGGTGTG-3′ and Rv: 5′-CCTTCCACAATGCCAAAGTT-3′) as a housekeeping gene.

### Chromatin immunoprecipitation PCR and sequencing

Sonicated covalently linked chromatin (∼200 bp), obtained using a chromatin immunoprecipitation (ChIP) assay kit (Merck Millipore, Watford, U.K.) and a Bioruptor plus (Diagenode, Seraign, Belgium), from 5 × 10^6^ BMMs stimulated for 30 min with 50 μg/ml 0–3hRP was immunoprecipitated with the following Abs: anti–p-CREB (clone 87G3), anti-CREB (clone 48H2) (both from Cell Signaling Technology), anti-RNA polymerase II (Merck Millipore), or anti-CD36 (Cayman Chemical, Ann Arbor, MI). DNA was recovered by reversing chromatin crosslinking and extracted using phenol-chloroform extraction. Extracted DNA was dissolved in 50 μl PCR-grade water and used for conventional PCR using four primer pairs expanding the +1 kb of the *Il10* promoter (first, Fw: 5′-AGAGGAGAGTTCTGGTGCCT-3′ and Rv: 5′-GGTGACTTCCGAGTCAGCAA-3′; second, Fw: 5′-AGAGGCCCTCATCTGTGGAT-3′ and Rv: 5′-GCAGAAGTTCATTCCGACCA-3′; third, Fw: 5′-TGTGGCTTTGGTAGTGCAAG-3′ and Rv: 5′-TGCTGCCTGCTCTTACTGAC-3′; and fourth, Fw: 5′-CTAGGAGCATGTGGCTCTGG-3′ and Rv: 5′-GTCTACCCGACAGCACAGAG-3′) or sent for sequencing. DNA libraries, sequencing, peak calling, and enrichment analysis were carried out by the Centre for Genomic Research at the University of Liverpool (Liverpool, U.K.). Detailed experimental design, protocol, data processing pipeline, and data have been deposited in National Center for Biotechnology Information’s Gene Expression Omnibus (45) and are accessible through accession number GSE64844 (http://www.ncbi.nlm.nih.gov/geo/query/acc.cgi?acc=GSE64844). Peaks with a <5% predicted false discovery rate and >65 peak scores [−10*log_10_(*p* value)] were annotated automatically with level of enrichment and used for subsequent analysis.

### Bioinformatics

Enriched peaks from ChIP sequencing data were visualized using the Integrative Genomics Viewer (46). Cytoscape (47, 48) and the app BinGO (49) were used to calculate gene ontology (GO) term enrichment, with a corrected *p* value cutoff of 0.05, and to generate GO term networks.

### Statistics

ANOVA and multiple comparisons tests (Bonferroni, Tukey, Sidak, and Dunnett) were performed to establish statistically significant differences between the groups (*p < 0.05, **p < 0.01, ***p < 0.001, ****p < 0.0001) using the software package GraphPad Prism (GraphPad Software, La Jolla, CA). Error bars represent SEM, based on biological replicates (i.e., pinnae from infected mice or separate bone marrow cell cultures from different animals).

## Results

### BMMs rapidly internalize *S. mansoni* cercarial E/S products and produce IL-10 in a TLR-dependent manner

**FIGURE 1.**
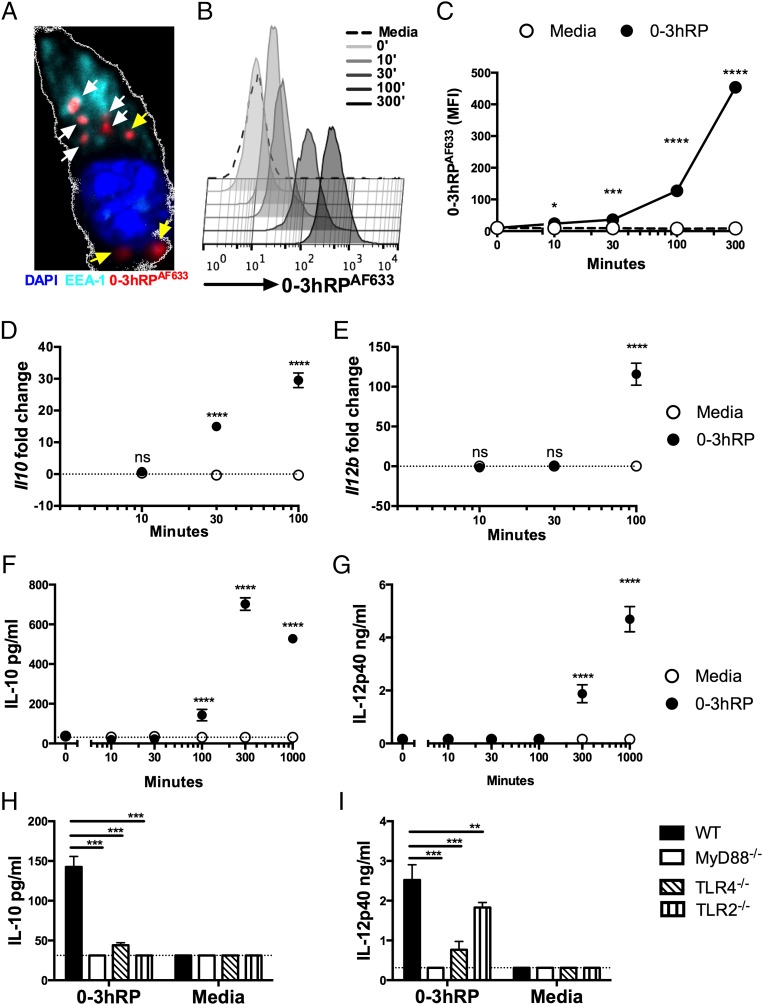
BMMs internalize cercarial E/S products and produce IL-10 in a TLR-dependent manner. (**A**) Representative confocal image (12 × 20 μm) of a BMM exposed to 50 μg/ml 0–3hRP^AF633^ (red) for 60 min, labeled with anti–EEA-1 Abs (cyan), and counterstained with DAPI (blue). Arrowheads indicate the location of 0–3hRP^AF633^ within EE-A^+^ (white) or EEA-1^−^ (yellow) endosomes. Cell membrane is shown in white. Representative overlaid flow cytometry histograms (**B**) and mean MFI ± SEM (**C**) of BMMs exposed to 50 μg/ml 0–3hRP^AF633^ (closed circles) or media control (open circles). Symbols are means of three technical replicates. Fold changes in transcript RNA (**D** and **E**) and protein levels of IL-10 and IL-12p40 (**F** and **G**) in cultures of WT BMMs exposed to 50 μg/ml 0–3hRP (closed circles) or corresponding media controls (open circles). Symbols represent mean from four biological replicates. (**H** and **I**) IL-10 and IL-12p40 produced by overnight cultures of WT, MyD88^−/−^, TLR2^−/−^, or TLR4^−/−^ BMMs exposed to 50 μg/ml 0–3hRP or media. Histogram bars are means for selected groups of WT and transgenic BMMs (four biological replicates). ANOVA and multiple comparisons tests (Bonferroni, Dunnett, and Sidak) were performed to examine statistically significant differences (C–G) between 0–3hRP–treated BMMs and corresponding media control at each time point or (H and I) between treated BMMs from different strains of mice. Dotted lines represent no fold changes in RNA levels (D and E) or lower detection limit of ELISA tests (F**–**I). Results are representative of three independent experiments. *p < 0.05, **p < 0.01, ***p < 0.001, ****p < 0.0001. ns, *p* > 0.05.

Discrete vesicles containing 0–3hRP^AF633^ were observed in BMMs at 60 min poststimulation, in both EEA-1^+^ (Fig. 1A, white arrows) and EEA-1^−^ vesicles (Fig. 1A, yellow arrows). Upon quantification, uptake of 0–3hRP^AF633^ between 10 and 300 min was revealed to be rapid but sustained, with median fluorescence intensity (MFI) values significantly greater in 0–3hRP^AF633^ exposed BMMs compared with corresponding media controls as early as 10 min poststimulation, rising thereafter out to 300 min (Fig. 1B, 1C; *p* < 0.05–0.0001). Similarly, increased levels of *Il10* transcript were detected rapidly after stimulation with 0–3hRP with significantly greater expression at 30 and 100 min after stimulation with 0–3hRP (Fig. 1D; *p* < 0.0001). In contrast, increased quantities of *Il12b* were only apparent by 100 min (Fig. 1E; *p* < 0.0001). The secretion of IL-10 protein by stimulated BMMs was more rapid (i.e., by 100 min, Fig. 1F) than the secretion of IL-12p40, which was only significantly elevated after 300 min (Fig. 1G).

IL-10 production in BMMs exposed to 0–3hRP was dependent on MyD88 signaling, but also on the presence of TLR2 and TLR4, as the production of this cytokine was reproducibly and greatly impaired in cells deficient for these molecules (Fig. 1H; *p* < 0.001). IL-12p40 production was similarly affected by the absence of these proteins (Fig. 1I; *p* < 0.001), although the absence of TLR2 appeared to have a more marginal role.

### BMMs stimulated with cercarial E/S products employ TLRs to activate ERK1/2, p38, and CREB

**FIGURE 2.**
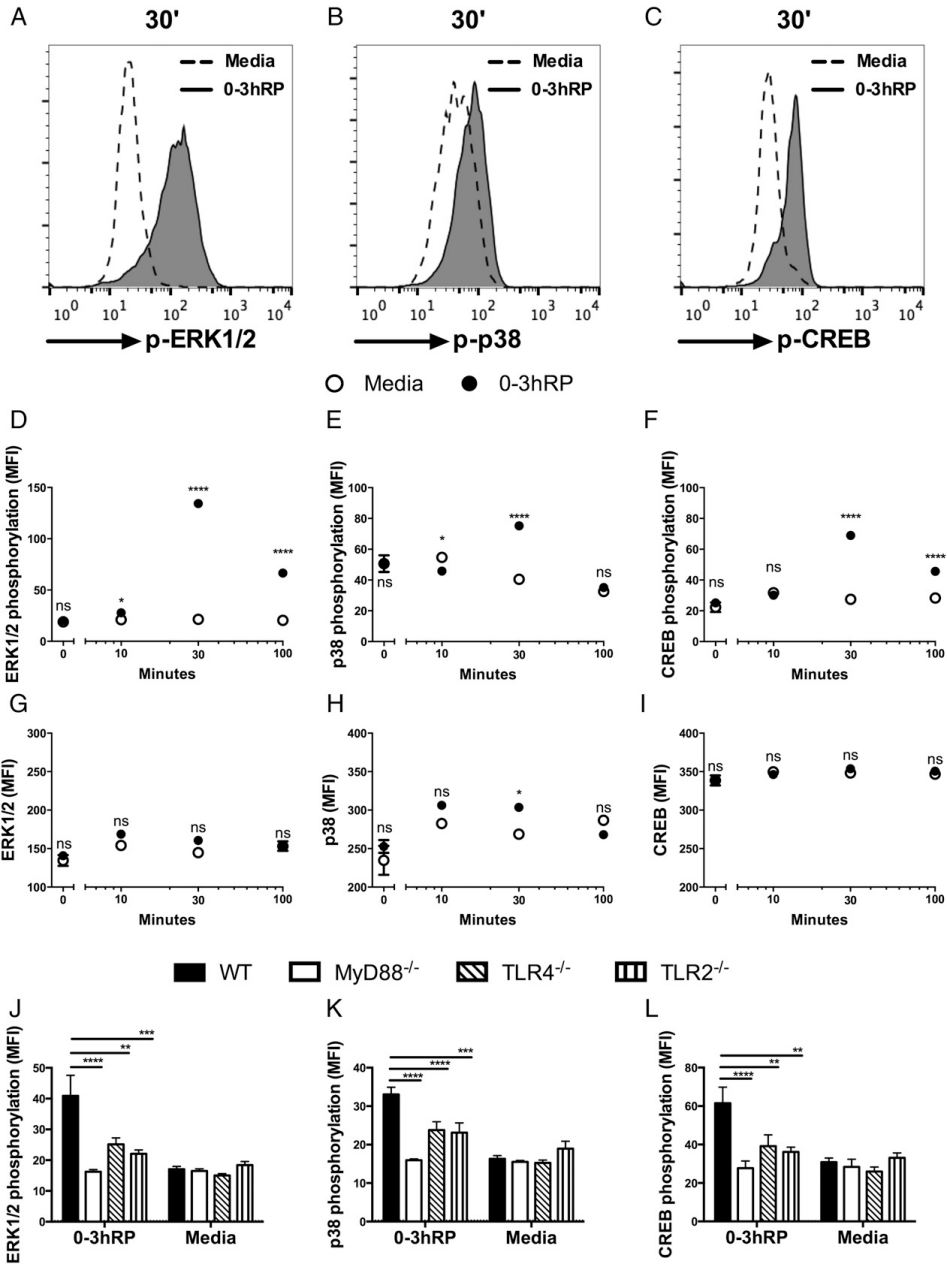
Cercarial E/S products trigger MAPK activation in BMMs in a TLR-dependent manner. Phosphorylation of MAPKs shown as representative overlaid flow cytometry histograms (**A**–**C**) and mean MFI ± SEM (**D**–**I**) for BMMs exposed to 50 μg/ml 0–3hRP (closed circles) or media control (open circles). Results are shown at 30 min (A–C and **J**–**L**) or 0–100 min (D–I). MFI values are shown for phosphorylation of ERK1/2, p38, and CREB (A–F and J–L) and for total MAPKs (G–I). Symbols and bars are means of four biological replicates and representative of three independent experiments. ANOVA and multiple comparisons tests (Sidak and Bonferroni) were performed to examine statistically significant differences between 0–3hRP–treated BMMs and corresponding media control at each time point (D–I) or between the means of selected groups of WT and transgenic BMMs (J and K) (four biological replicates). *p < 0.05, **p < 0.01, ***p < 0.001, ****p < 0.0001. ns, *p* > 0.05.

As TLR-driven IL-10 production in BMMs occurred rapidly (30 min) after stimulation with 0–3hRP, early phosphorylation events in these cells were investigated. A kinase profiler array revealed that several proteins became phosphorylated after stimulation including ERK1/2, p38, and CREB (Supplemental Fig. 1, white arrows). Although limited activation of these kinases was seen after 5 min (Supplemental Fig. 1, *top panel*), all three were abundantly phosphorylated after 30 min (Supplemental Fig. 1, *middle panel*) and were still detectable after 60 min (Supplemental Fig. 1, *bottom panel*). Significantly greater levels of IL-10 were detected in culture supernatants of BMMs treated with 0–3hRP compared with TLR agonists LPS, polyinosinic-polycytidylic acid, and Pam3CSK4 (Supplemental Fig. 2A, *p* < 0.05–0.001), whereas significantly lower levels of IL-12p40 were obtained after 0–3hRP stimulation (Supplemental Fig. 2A, *p* < 0.001–0.0001). Moreover, both ERK2 and CREB phosphorylation was significantly elevated in 0–3hRP stimulated BMMs compared with LPS (Supplemental Fig. 2C–E; *p* < 0.0001), whereas phosphorylation of p38 was equivalent (Supplemental Fig. 2C, 2F). Phosphorylation dynamics of all three kinases were closely examined using flow cytometry (Fig. 2A–C), as this technology has been widely used to study phosphorylation of proteins at a cellular level permitting truly quantitative and statistical analysis, while avoiding artifacts introduced by enzymatic amplification and dead cells (50, 51). Our data expressed as mean MFI ± SEM using four biological replicates representative of three independent experiments revealed that all three kinases shared similar activation profiles, with maximal phosphorylation detected 30 min after stimulation with 0–3hRP (Fig. 2D–F; *p* < 0.0001), and this was verified for ERK1/2 by Western blot (Supplemental Fig. 2G). In comparison, no significant changes were detected in the levels of total protein (Fig. 2G–I). Phosphorylation of ERK1/2, p38, and CREB was entirely dependent on the availability of MyD88 and was partly dependent on the presence TLR2 and TLR4 (Fig. 2J–L; *p* < 0.01–0.0001). Consequently, 0–3hRP–driven activation of these three MAPK was dependent on TLR signaling, as previously shown for IL-10 production.

Both p65 and p105 components of the NF-κB system were likewise phosphorylated after exposure to 0–3hRP (Supplemental Fig. 3A, 3B), although the activation profile did not resemble that of ERK1/2, p38, and CREB (Supplemental Fig. 3D, 3E). In contrast, although RSK became phosphorylated in 0–3hRP–stimulated BMMs (Supplemental Fig. 3C), it shared the same activation profile as ERK1/2, p38, and CREB (Supplemental Fig. 3F) and did not exhibit significant changes in the total levels of RSK protein (Supplemental Fig. 3G).

**FIGURE 3.**
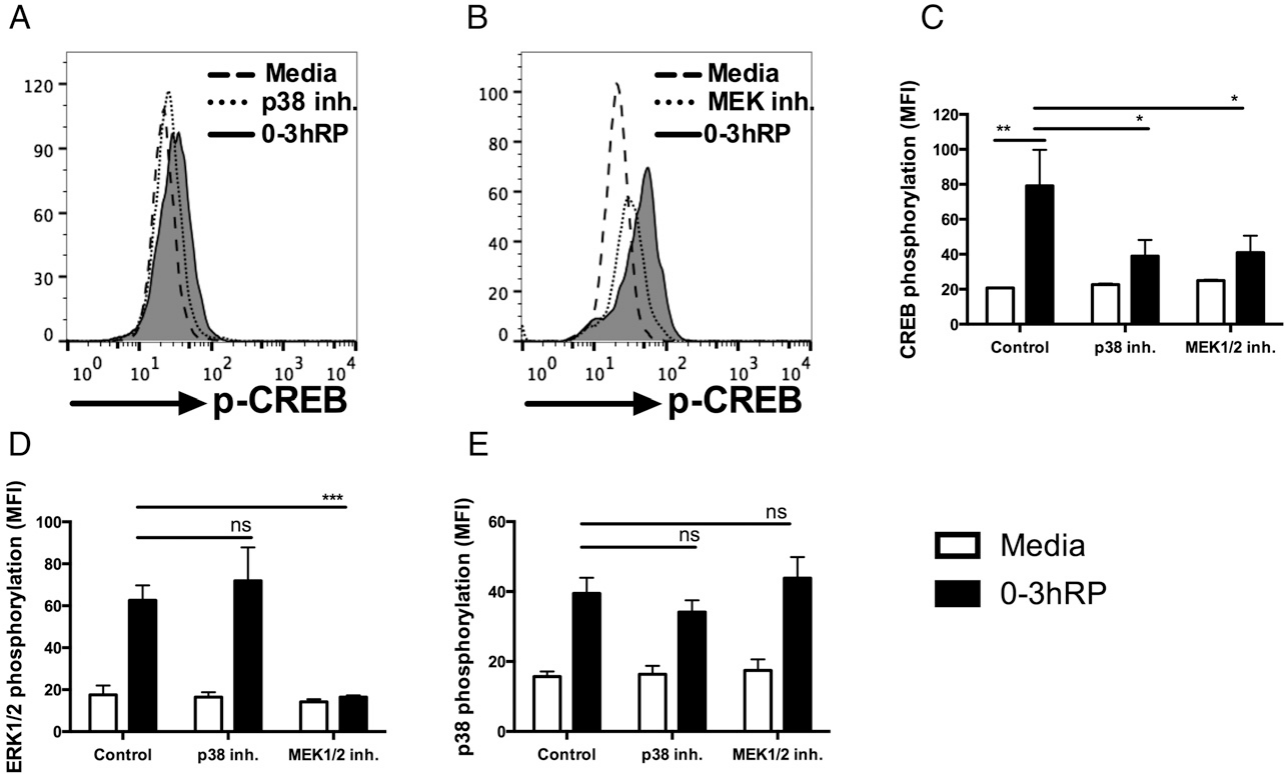
CREB is downstream from p38 and ERK1/2. Representative overlaid flow cytometry histograms (**A** and **B**) and mean MFI + SEM (**C**–**E**) of BMMs treated with p38 inhibitor (SB203580, 1 μmol) or MEK inhibitor (U0126, 10 μmol) for 2 h and then exposed for 30 min to 50 μg/ml 0–3hRP (closed bars) or media control (open bars). Cells were labeled with Abs against p-CREB (C), p-ERK1/2 (D), or p-p38 (E). Bars represent means of three biological replicates. ANOVA and Tukey multiple comparisons test were performed to examine statistically significant differences between the means of selected groups. Results are representative of four independent experiments. *p < 0.05, **p < 0.01, ***p < 0.001. ns, *p* > 0.05.

Chemical inhibition of p38 prior to stimulation of BMMs with 0–3hRP resulted in reduced phosphorylation of CREB (Fig. 3A, 3C; *p* < 0.05), similar to the effects after the chemical inhibition of MEK1/2 (Fig. 3B, 3C; *p* < 0.05), indicating that both p38 and MEK/ERK are upstream of CREB. In contrast, inhibition of p38 had no effect on the phosphorylation of ERK1/2 (Fig. 3D), whereas MEK1/2 inhibitor completely ablated phosphorylation of ERK1/2 (Fig. 3D; *p* < 0.001). Inhibition of p38 using SB203580 is known to have no effects over the levels of phosphorylated p38 (52), in line with our own finding (Fig. 3E). Inhibition of MEK1/2 also had no effect on p38 phosphorylation (Fig. 3E). Thus, off-target effects of p38 and MEK1/2 inhibitors did not account for the observed reductions in CREB phosphorylation, and consequently, it was concluded that CREB activation was a result of p38 and MEK/ERK activation. Inhibition of p38 had no effect on the phosphorylation of RSK in 0–3hRP–stimulated BMMs compared with resting cells, whereas inhibition of MEK/ERK resulted in a significant reduction (Supplemental Fig. 3H; *p* < 0.0001), indicating that MEK/ERK are upstream of RSK.

### ERK1/2, p38, and CREB control IL-10 and limit IL-12 in BMMs stimulated with cercarial E/S products

**FIGURE 4.**
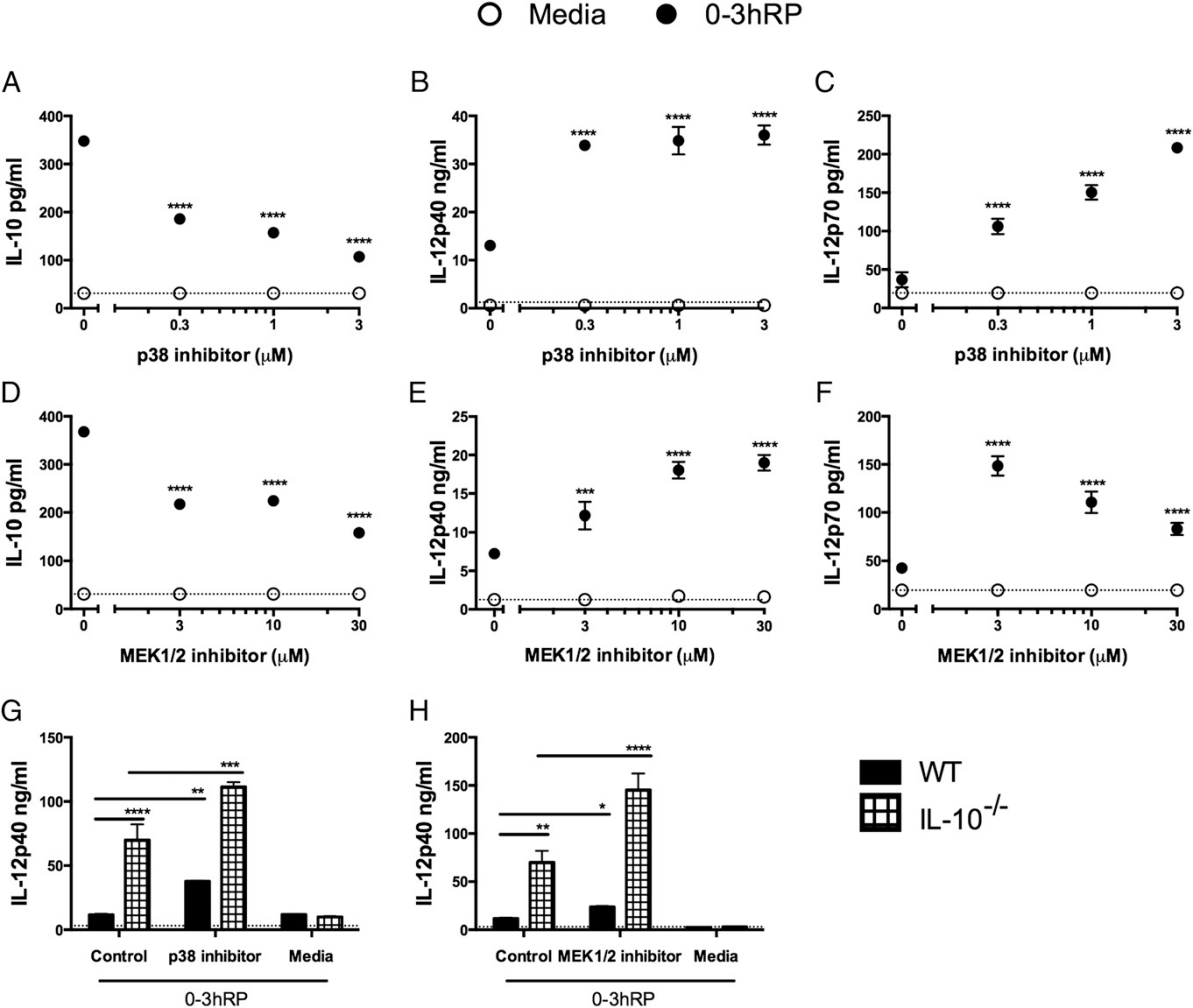
MAPK activation induces IL-10 production, while limiting IL-12. BMMs from WT (**A**–**F**) and IL-10^−/−^ (**G** and **H**) mice were treated with p38 inhibitor (SB203580, 0.3–3 μmol) (A**–**C and G) or MEK inhibitor (U0126, 3–30 μmol) (D**–**F and H) for 2 h and then exposed overnight to 50 μg/ml 0–3hRP (closed circles) or media control (open circles). Culture supernatants were tested for the presence of IL-10 (A and D), IL-12p40 (B, E, G, and H), and IL-12p70 (C and F). Symbols and bars are means of four biological replicates. ANOVA and multiple comparisons tests (Dunnett and Sidak) were performed to examine statistically significant differences (A–F) between 0–3hRP–treated BMMs and corresponding media control at each dose of the inhibitor or between the means of WT and IL-10^−/−^ BMMs (G and H) (four biological replicates). Dotted lines represent lower detection limit in ELISA tests. Results are representative of three independent experiments. *p < 0.05, **p < 0.01, ***p < 0.001, ****p < 0.0001. ns, *p* > 0.05.

The link between MAPK phosphorylation and cytokine production was demonstrated because chemical inhibition of p38 resulted in a significant dose-dependent decrease in IL-10 production by BMMs exposed to 0–3hRP (Fig. 4A; *p* < 0.0001), whereas there was a significant increase in the levels of IL-12p40 and IL-12p70 (Fig. 4B, 4C; *p* < 0.0001). Inhibition of MEK1/2 also resulted in a marked reduction in the levels of IL-10 (Fig. 4D; *p* < 0.0001), accompanied by significant increases in the production of IL-12p40 and IL-12p70 (Fig. 4E, 4F; *p* < 0.0001). Consequently, IL-10 production in BMMs stimulated with 0–3hRP is controlled by ERK1/2 and p38.

Increased production of IL-12 by 0–3hRP–stimulated BMMs treated with inhibitors for p38 and MEK1/2 could be a result of the corresponding reduction in IL-10 production; however, IL-10^−/−^ BMMs exposed to 0–3hRP and treated with p38 inhibitor (Fig. 4G), or MEK1/2 inhibitor (Fig. 4H), exhibited significantly increased levels IL-12p40 compared with uninhibited controls (*p* < 0.001–0.0001). Thus, IL-12 production is limited by the activation of p38 and ERK1/2 independently of IL-10. In addition, BMMs deficient for TPL2, which is required for TLR-driven ERK1/2 phosphorylation (40, 53), failed to phosphorylate ERK1/2 in response to 0–3hRP (Supplemental Fig. 3I), but exhibited increased levels of IL-12p40 (Supplemental Fig. 3J; *p* < 0.0001), concurrent with decreased production of IL-10 (Supplemental Fig. 3K; *p* < 0.05), further confirming the role of ERK1/2 in limiting IL-12 production.

**FIGURE 5.**
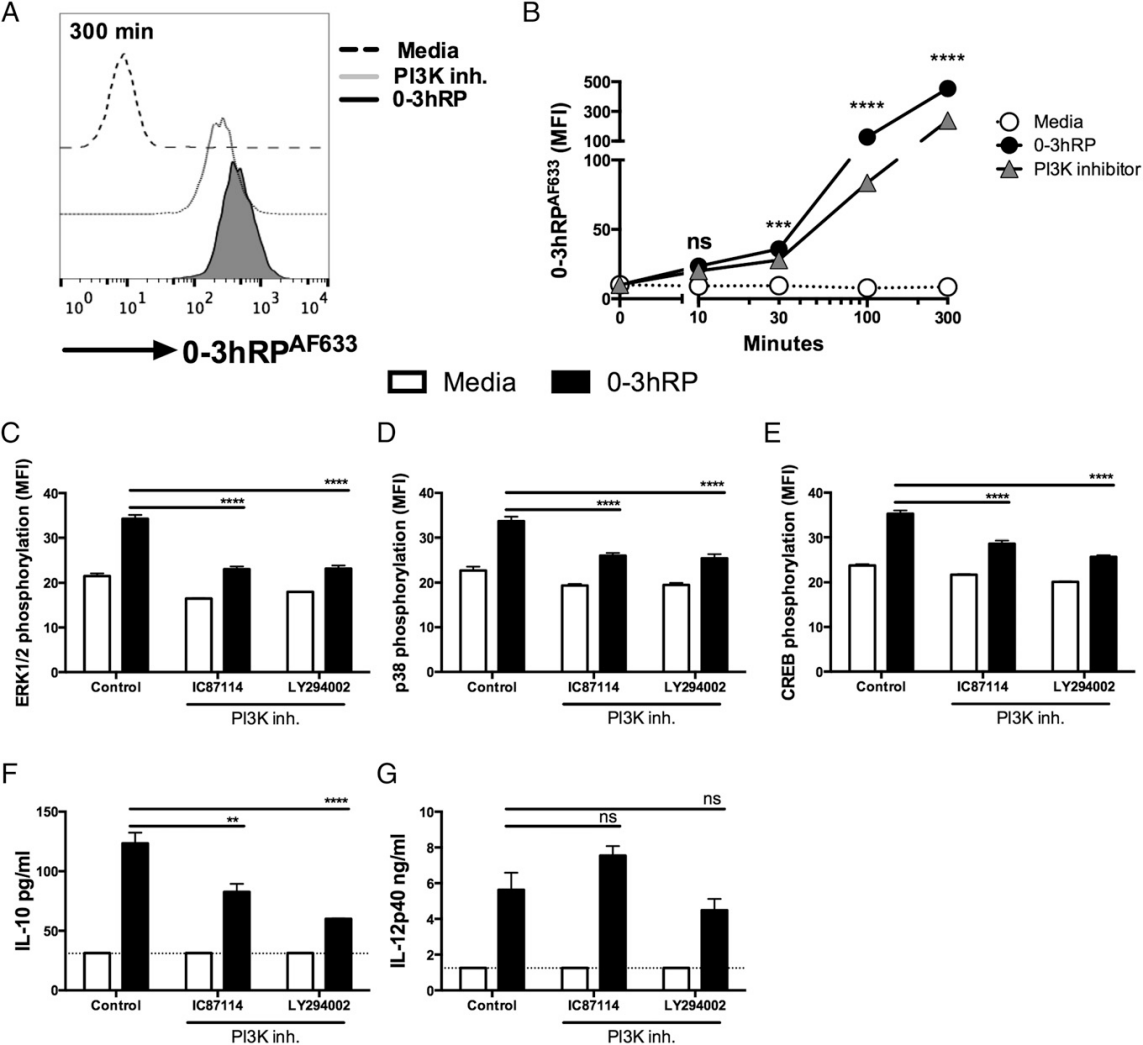
Uptake of 0–3hRP and PI3K signaling is required for full MAPK activation and IL-10 production by BMMs. Representative overlaid flow cytometry histograms (**A**) and mean MFI ± SEM (**B**) of BMMs pretreated for 2 h with PI3K inhibitor (LY294002; 25 μmol; gray line/squares) or left untreated (circles) and then exposed to 50 μg/ml 0–3hRP^AF633^ (closed circles) or media control (dashed line, open circles) for 0–300 min. Symbols represent means of three biological replicates. (**C–G**) BMMs pretreated for 2 h with PI3K inhibitors LY294002 or IC87114 or left untreated and then exposed to 0–3hRP for 30 min (closed bars) or media control (open bars). Cells were labeled with Abs against p-ERK1/2 (C), p-p38 (D), or p-CREB (E). Bars represent mean MFI + SEM of three biological replicates. Supernatants from BMM cultures treated with PI3K inhibitors LY294002 and IC87114 (F and G) and stimulated with 0–3hRP (closed bars) or media control (open bars) were tested for the presence of IL-10 and IL-12p40. Bars represent means of three biological replicates. ANOVA and multiple comparisons tests (Sidak and Dunnett) were performed to examine statistically significant differences (B**–**G) between BMMs exposed to 0–3hRP only versus cells exposed 0–3hRP + inhibitor. Dotted lines are the lower detection limit in ELISA tests. Results are representative of four independent experiments. **p < 0.01, ***p < 0.001, ****p < 0.0001. ns, *p* > 0.05.

IL-10 production linked to rapid MAPK activation could be related to the quick internalization of 0–3hRP by BMMs. Indeed, uptake of 0–3hRP^AF633^ was significantly impaired from 100 min with PI3K inhibitors (Fig. 5A, 5B). Decreased phosphorylation of ERK1/2, p38, and CREB in BMMs stimulated with 0–3hRP for 30 min was recorded following inhibition using a p110δ-specific inhibitor (IC87114) or with a PI3K family inhibitor (LY294002) (Fig. 5C–E; *p* < 0.0001). Both PI3K inhibitors greatly reduced the production of IL-10 by stimulated BMMs (Fig. 5F; *p* < 0.001–0.0001), but had no effect upon the production of IL-12p40 (Fig. 5G).

### CREB is recruited to the IL-10 promoter in BMMs exposed to 0–3hRP

As BMMs appear likely to use TLR2 and TLR4 to recognize 0–3hRP, resulting in MyD88-mediated activation of MEK/ERK and p38, which subsequently converge in the phosphorylation of the transcription factor CREB, it is possible that CREB is recruited to the IL-10 promoter, where it participates in the initiation of *Il10* mRNA synthesis.

**FIGURE 6.**
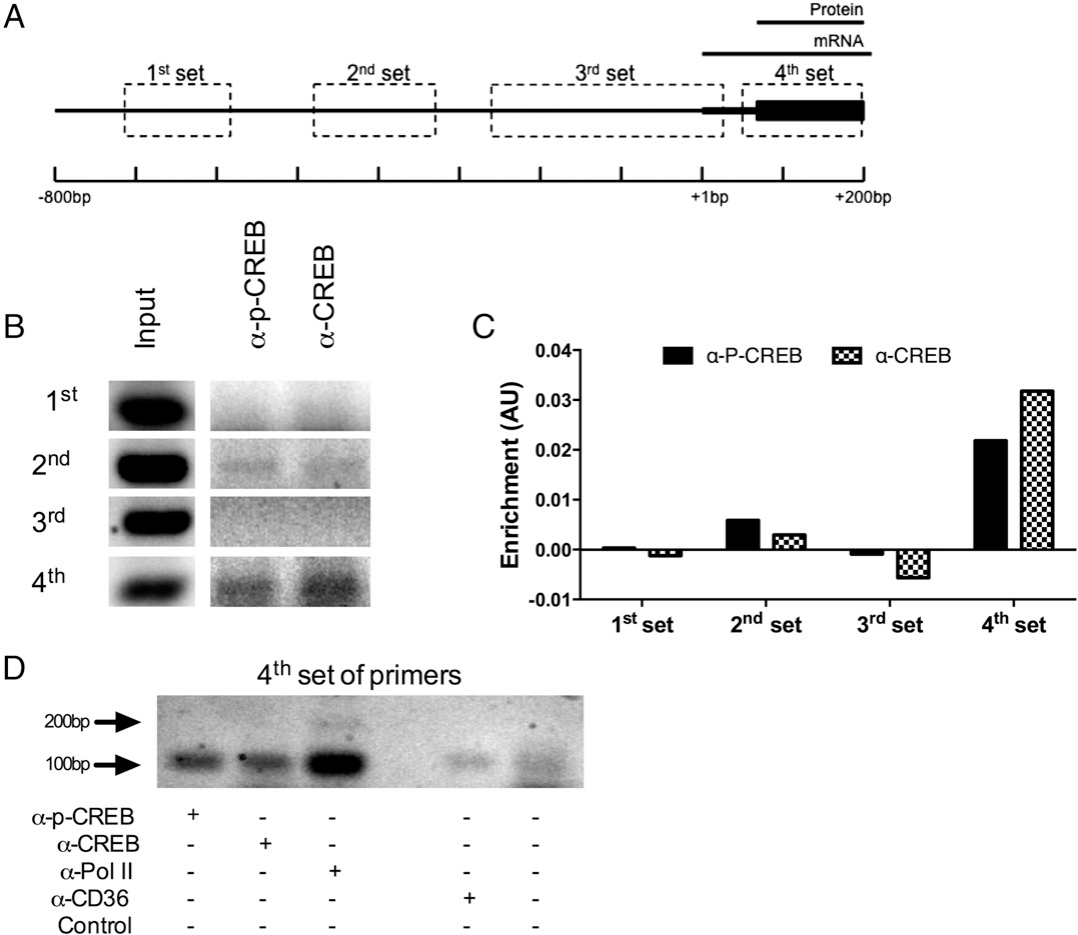
CREB is recruited to a specific regulatory element of the *Il10* promoter. (**A**) Scan strategy used to determine binding site in the *Il10* promoter for CREB using sets of primers to amplify four DNA fragments between 100 and 300 bp (dotted boxes). Transcription and translation start sites are indicated above diagram. Agarose gel of amplified DNA fragments from *Il10* promoter using all designed primers (**B**) and enrichment of promoter regions based on densitometry analysis of PCR results (**C**). Sonicated chromatin from BMMs exposed to 0–3hRP for 30 min was precipitated using Abs against p-CREB (black bar), total CREB (checked bar), plus [in (**D**)] control Abs for RNA polymerase II (Pol II) and CD36, alongside no Ab control. Results are representative of four independent experiments. AU, arbitrary units.

Using four sets of primers mapping the IL-10 promoter region (Fig. 6A) to analyze fragmented chromatin obtained from BMMs stimulated with 0–3hRP precipitated with Abs against p-CREB and total CREB, a potential binding site for CREB was found within the fourth region (Fig. 6B), which was the most enriched portion of the promoter detected (Fig. 6C). Moreover, chromatin precipitated with anti–Pol II Abs also produced a strong signal with the fourth set of primers because *Il10* is transcriptionally active after 30 min of 0–3hRP stimulation (Fig. 6D). Much fainter signals were detected from negative controls using Abs against nonnuclear CD36 and no Ab control for all the primer pairs, as shown in this study for the fourth set (Fig. 6D). Consequently, p-CREB is recruited to the IL-10 promoter in BMMs 30 min after exposure to 0–3hRP, where it is likely to modulate the transcription of IL-10.

### CREB regulates a network of genes in BMMs exposed to 0–3hRP

**FIGURE 7.**
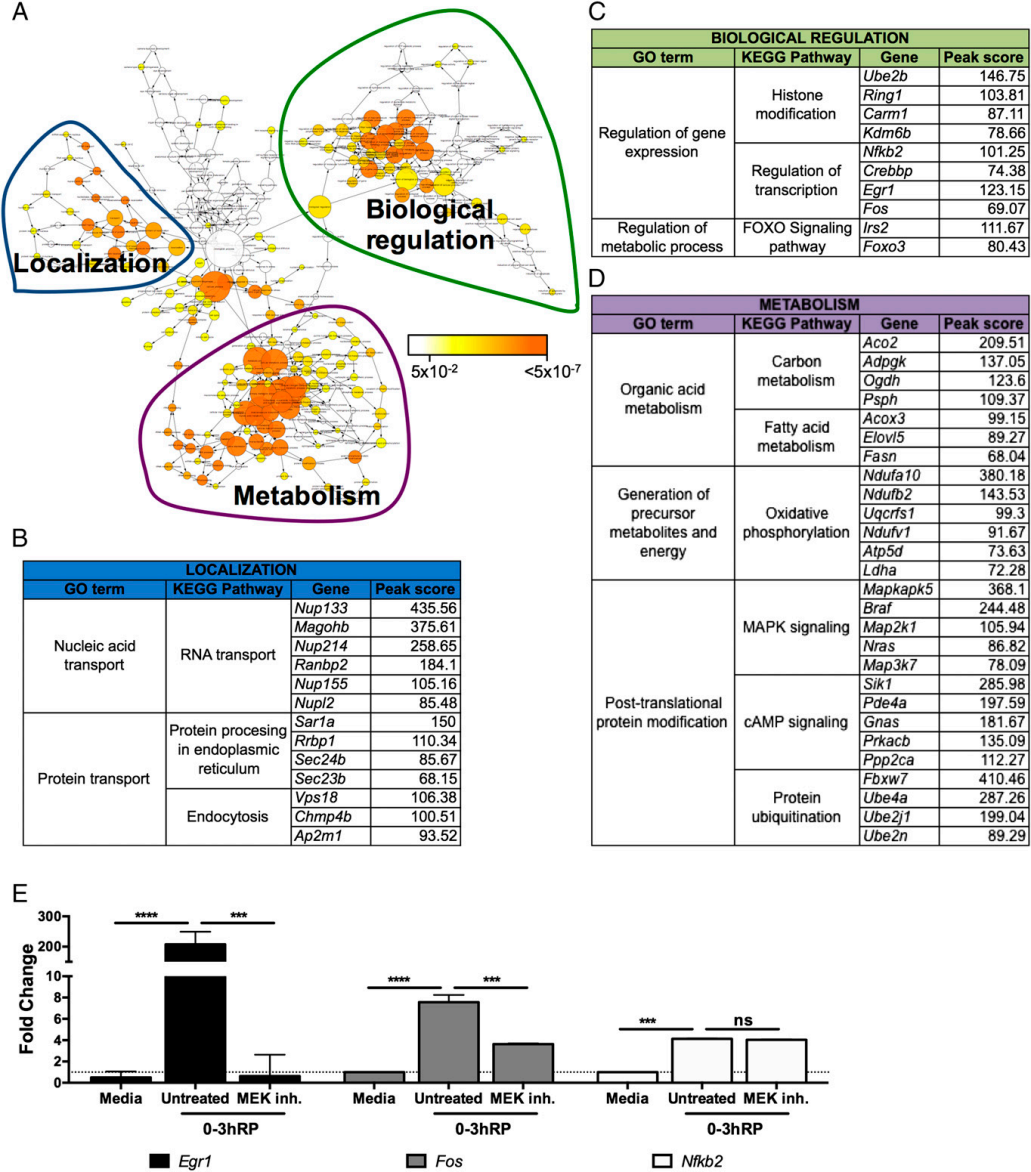
TLR-activated CREB regulates an extensive network of genes in BMMs stimulated with cercarial E/S products. GO term enrichment network divided into three clusters from enriched peaks + peak scores [−10*log_10_(*p* value)] from sequenced fragments of sonicated chromatin from BMMs exposed 0–3hRP precipitated using an Ab against CREB calculated against input control. (**A**) Significantly enriched GO terms (*p* < 0.05) colored based on significance (according to intensity of color gradient). Node size is representative of the number of genes mapping to each term. Significantly enriched genes within “localization” (**B**), “biological regulation” (**C**), and “metabolism” (**D**) clusters, presented according to their GO term and KEGG pathway. (**E**) Mean fold change + SEM for selected RNA transcript levels obtained from BMMs pretreated with MEK inhibitor or left untreated and then exposed to 0–3hRP or media control. Bars represent means of three technical replicates. Statistically significant differences between the means of selected groups were determined using ANOVA and Tukey multiple comparisons test. Dotted lines represent no-fold change in the levels of mRNA. ***p < 0.001, ****p < 0.0001. ns, *p* > 0.05.

CREB-precipitated chromatin fragments were sequenced, and those that were significantly enriched peaks (peak score >65) based on input with a false discovery rate <5% were identified [raw data available at the National Center for Biotechnology Information’s Gene Expression Omnibus (45), accession number GSE64844]. After peak calling, 654 genes proximal to identified peaks were analyzed for significant GO term enrichment (*p* < 0.05), which were then used to create a network of “Biological process” GO terms (Fig. 7A). Three discrete GO term clusters were defined—“metabolism,” “localization,” and “biological regulation” (Fig. 7B–D)—and GO terms within selected nodes were further annotated with relevant Kyoto Encyclopedia of Genes and Genomes pathways. The “localization” cluster included genes involved in RNA transport by forming part of the nuclear pore complex (*Nup133*), protein processing (*Sar1a*), or endocytosis (*Vps18*) (Fig. 7C). Moreover, the “biological regulation” cluster included genes involved in histone modification (i.e., *Ube2b* and *Ring1*), as well as transcription factors (i.e., *Egr1*, *Fos*, and *Nfkb2*) (Fig. 7C). The expression of this last group of genes was confirmed in BMMs exposed to 0–3hRP for 30 min, which significantly increased the levels of these transcripts compared with media controls (Fig. 7E; *p* < 0.0001). In the case of *Egr1* and *Fos*, expression of these genes was partly dependent upon ERK1/2 phosphorylation as chemical inhibition of MEK1/2 significantly reduced the levels of both transcripts (Fig. 7E; *p* < 0.001). Thus, activation of CREB in 0–3hRP–stimulated BMMs, through MEK/ERK and potentially p38, regulates the expression of a network of genes involved in regulation of transcription.

**FIGURE 8.**
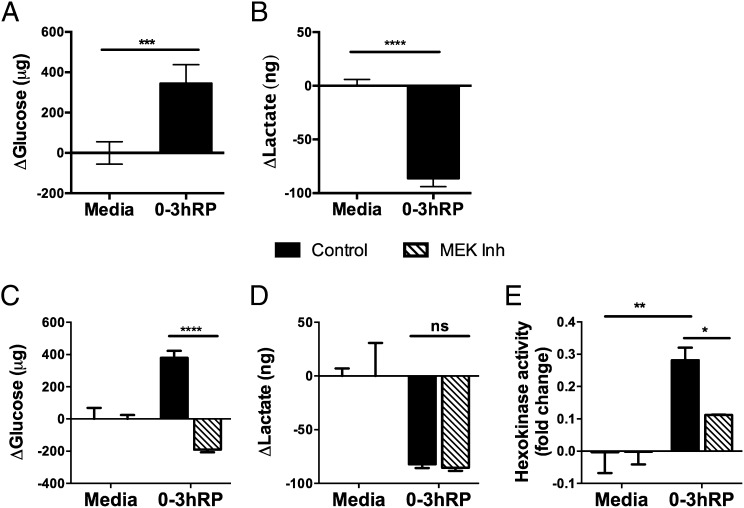
0–3hRP stimulation increases oxidative phosphorylation over anaerobic glycolysis in BMMs downstream of MEK1/2. Culture supernatants from BMMs exposed for 18 h to 50 μg/ml 0–3hRP or left unstimulated (media) were tested for the amount of remaining glucose (**A**) and lactate production (**B**). (**C** and **D**) BMMs were treated for 2 h with MEK1/2 inhibitor U0126 (10 μmol) and then exposed for 18 h to 50 μg/ml 0–3hRP or left unstimulated (media). Culture supernatants were tested for the amount of remaining glucose (C) and lactate production (D). Additionally, stimulated BMMs were lysed and hexokinase activity measured (**E**). Bars represent the mean difference + SEM of the amount of each metabolite (*n* = 3) (A**–**D) or mean fold changes + SEM (E) in hexokinase activity (*n* = 3). In all cases, media was used as a reference point arbitrarily set to 0. (A and B) Unpaired two-tailed *t* tests were performed to examine differences between means of 0–3hRP–treated cells compared with media. (C and D) ANOVA and Sidak multiple comparisons test were performed to examine statistically significant differences between control BMMs stimulated with 0–3hRP (black bars) compared with BMMs treated with MEK1/2 inhibitor then stimulated with 0–3hRP (hatched bars). *p < 0.05, **p < 0.01, ***p < 0.001, ****p < 0.0001. ns, *p* > 0.05.

Notably, the “Metabolic process” GO term was the most enriched and contained 48.02% of all annotated genes. The metabolism cluster, which includes this term, encompassed genes involved with the initiation of glycolysis (*Adpgk*), key steps in the tricarboxylic acid cycle (*Aco2*) and during oxidative phosphorylation (*Ndufa10*), whereas this GO term cluster also included genes involved in MAPK signaling and notably some upstream from ERK1/2 (i.e., *Braf*, *Nras*, and *Map2k1*) (Fig. 7B). Indeed, BMMs exposed to 0–3hRP depleted significantly more glucose from culture medium than resting macrophages (Fig. 8A; *p* < 0.001), although 0–3hRP–stimulated cells produced significantly less lactate (Fig. 8B; *p* < 0.0001). Although glucose is used as fuel in both oxidative phosphorylation and anaerobic glycolysis, lactate is only the product of the latter, supporting our hypothesis that BMMs exposed to 0–3hRP rely on oxidative phosphorylation. Treatment of 0–3hRP–stimulated BMMs with MEK1/2 inhibitor revealed that glucose consumption (Fig. 8C), but not lactate production (Fig. 8D), is regulated by 0–3hRP–triggered ERK1/2 phosphorylation. Indeed, BMMs treated with MEK1/2 inhibitor consumed significantly less glucose than their uninhibited counterparts (Fig. 8C; *p* < 0.0001). In accordance with observed glucose uptake, hexokinase activity was also greatly enhanced in macrophages exposed to 0–3hRP (Fig. 8E; *p* < 0.01) and was reduced after MEK1/2 inhibition (Fig. 8E; *p* < 0.05). Consequently, energy metabolism in BMMs following 0–3hRP stimulation, which relies heavily on oxidative phosphorylation, is modulated by TLR mediated activation of ERK1/2.

### Resident skin macrophages in vivo rapidly produce IL-10 following infection with *S. mansoni* cercariae

**FIGURE 9.**
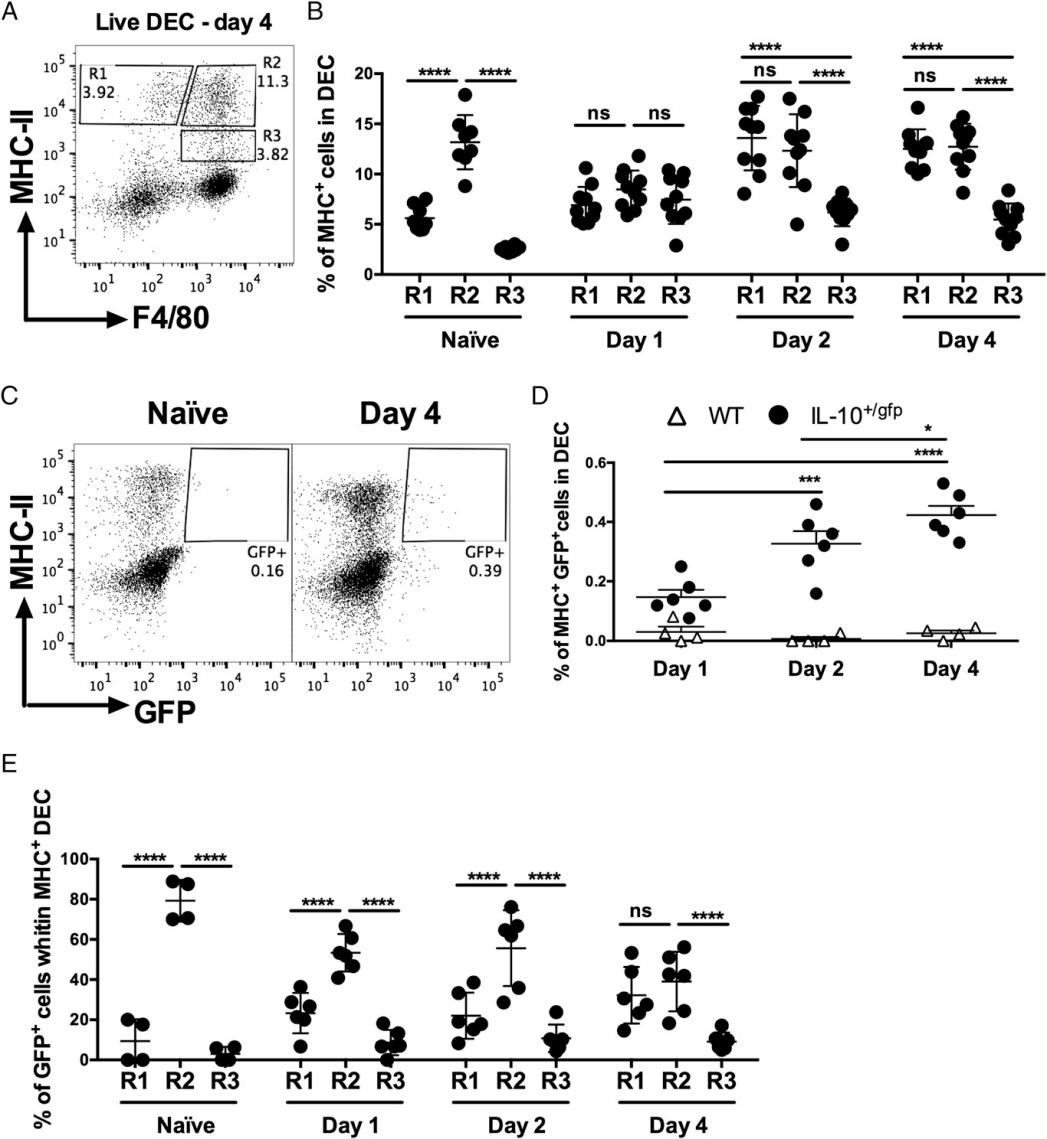
IL-10 is produced in the skin by two different monocyte populations following *S. mansoni* cercariae infection. Representative flow cytometry dot plots (**A** and **C**) and mean percentage ± SEM (**B**, **D**, and **E**) of DEC according to expression of MHC-II and F4/80. Gates show R1 (F4/80−MHC-II^high^), R2 (F4/80^+^MHC-II^high^), and R3 (F4/80^+^MHC-II^mid^) monocytes. MHC-II^+^ DEC (A and B) and IL-10/GFP^+^ MHC-II^+^ DEC (C–E), recovered from naive animals or specified days postinfection of WT or IL-10^+/GFP^ mice. (E) IL-10/GFP^+^ MHC-II^+^ DEC separated according to expression of F4/80 as in (A). Symbols are values for cells obtained from individual naive/infected mice. Horizontal bars are the means ± SEM. *n* = 4–10 pinnae. ANOVA and Tukey multiple comparisons test show statistically significant differences between the means of indicated groups. *p < 0.05, ***p < 0.001, ****p < 0.0001. ns, *p* > 0.05.

We attempted to address the relevance of the in vitro observations, reported above in the context of in vivo conditions following infection of a mammalian host, using skin exposed to *S. mansoni* cercariae. A well-characterized percutaneous infection model (9) was employed to obtain DEC from infected pinnae to study IL-10 production by monocytes at early time points. Three discrete monocyte populations (R1: F4/80^−^MHC-II^high^; R2: F4/80^+^MHC-II^high^; and R3: F4/80^+^MHC-II^mid^) were evident within DEC based on the expression of F4/80 and MHC-II (Fig. 9A). F4/80^+^MHC-II^high^ tissue macrophages (R2) were the most abundant MHC-II^+^ population in naive skin (Fig. 9B). However, by day 1 postinfection, the proportion of all three populations was not significantly different (Fig. 9B), although by days 2 and 4, F4/80^−^MHC-II^high^ DC (R1) and F4/80^+^MHC-II^high^ tissue macrophages (R2) were equally abundant, and both were significantly more abundant than F4/80^+^MHC-II^mid^ macrophages (R3) (Fig. 9B; *p* < 0.0001).

**FIGURE 10.**
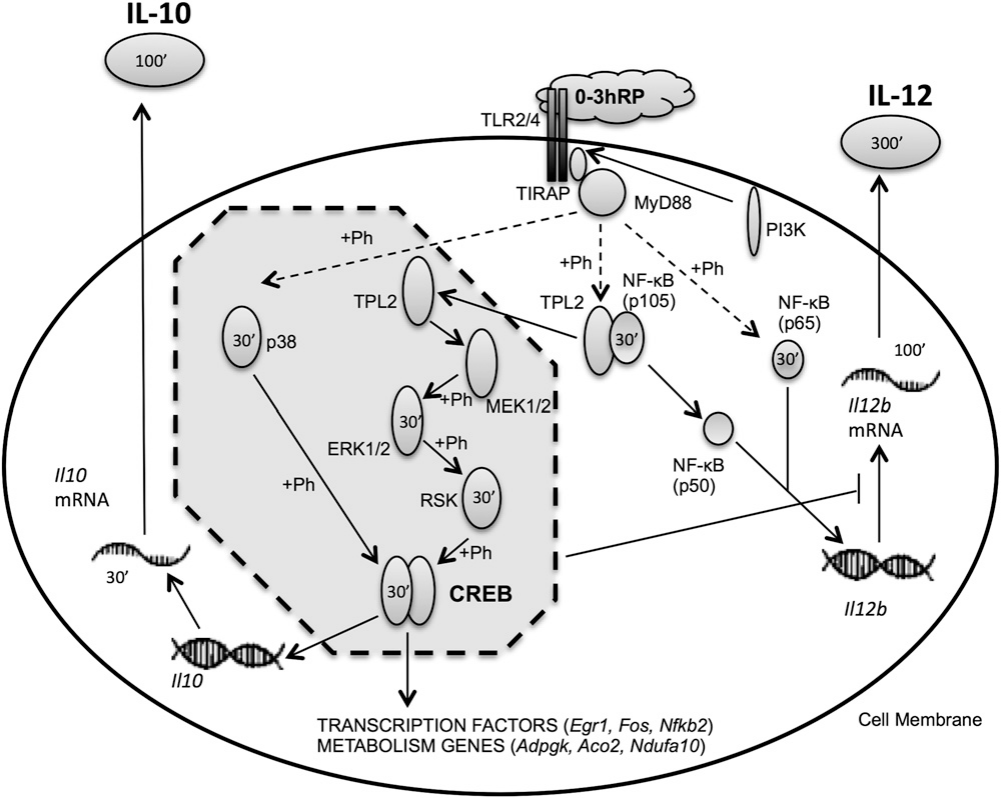
Hypothetical model of the molecular events leading to the production of IL-10 in macrophages exposed to *S. mansoni* cercarial E/S products. *S. mansoni* cercarial E/S products are likely to engage TLR2 and TLR4 in macrophages, after which TIRAP, and consequently MyD88, would be recruited to the activated receptors in a PI3K-dependent manner. These events trigger the phosphorylation (+Ph) of p38 and NF-κB proteins p65 and p105. The latter releases TPL2, thus activating the MEK/ERK/RSK cascade. The two axes of this signaling pathway (enclosed in a dotted line) converge on the phosphorylation of CREB, which is recruited to the *Il10* promoter, leading to the production of IL-10, and also modulates the expression of genes associated with metabolism and several transcription factors. Phosphorylated p65 and p50, the degradation product of p105, can form a heterodimer, which modulates the transcription of IL-12, a process negatively impacted by the mechanism that leads to IL-10 production (enclosed in dotted lines). Peak phosphorylation times in minutes are given for several kinases, as well as the initial detection times for the mRNA and protein of cytokines.

IL-10–producing MHC-II^+^ cells based on GFP expression increased steadily from day 1 to day 4 after *S. mansoni* infection (Fig. 9C, 9D), with most MHC-II^+^IL-10^+^ DEC detected on the fourth day after cercarial penetration (*p* < 0.05–0.0001). Based upon expression of F4/80 and MHC-II (as in Fig. 9A), IL-10^+^ F4/80^+^MHC-II^high^ tissue macrophages (R2) were more abundant than any other IL-10^+^ MHC-II^+^ cells in naive DEC, as well as on days 1 and 2 after *S. mansoni* infection (Fig. 9E; *p* < 0.0001). By day 4, the proportion of IL-10^+^ F4/80^−^MHC-II^high^ DC (R1) and IL-10^+^ F4/80^+^MHC-II^high^ tissue macrophages (R2) was equivalent and significantly higher than IL-10^+^ F4/80^+^MHC-II^mid^ macrophages (R3) (Fig. 9E; *p* < 0.0001). Consequently, penetration of *S. mansoni* cercariae, and their subsequent release of E/S products, triggers IL-10 production in vivo by two types of monocytes in the skin, with tissue macrophages rapidly producing IL-10 after exposure to the parasite. Thus, we propose a hypothetical model (Fig. 10) based on our in vitro studies, by which tissue macrophages produce IL-10 after *S. mansoni* infection.

## Discussion

BMMs stimulated with 0–3hRP exhibit an activation state characterized by the production of abundant anti-inflammatory IL-10, which followed the activation of several MAPKs downstream of both TLR2 and TLR4. The activation profile of these kinases matched the transcription of *Il10* and resulted from recruitment of CREB to the promoter region of *Il10*. Intriguingly, phosphorylation of CREB in BMMs stimulated with 0–3hRP was linked to altered metabolic processes, particularly glycolysis, the tricarboxylic acid cycle, and oxidative phosphorylation, suggesting that macrophages require specific metabolic programs to respond to 0–3hRP. Finally, we observed that during the first days postinfection of the skin by *S. mansoni* cercariae, skin tissue macrophages were the initial and dominant myeloid source of IL-10, and we propose that when exposed to cercarial E/S products during infection, they are likely to employ the mechanism we unraveled in vitro to produce IL-10.

Several helminth products are known to be associated with stimulation of various TLRs (54–57), whereas *Schistosoma* Ags have been reported to interact with specific TLRs present on mononuclear phagocytes (32, 34, 58, 59). However, few of these studies describe the signaling pathways triggered in host cells or indeed link them to the production of specific cytokines (58–60). In the current study, we show that in BMMs stimulated with 0–3hRP, both TLR2 and TLR4 are necessary for the production of IL-10 in a MyD88-dependent manner. As *S. mansoni* cercarial E/S products are a complex mixture of glycosylated proteins (25, 27), it is possible that TLR2 and TLR4 each recognize different components of 0–3hRP, thus explaining the differences in IL-12 production observed in the absence of TLR2 versus TLR4. However, the absence of both receptors had a comparable effect on the production of IL-10 and the phosphorylation of p38, ERK1/2, and CREB. The potential mechanism that links IL-10 production to TLR ligation in BMMs exposed to cercarial E/S products (hypothesis summarized in Fig. 10) depends on two linked MAPK cascades. On one hand, p38 was phosphorylated, an event often reported downstream of TLRs (61, 62), whereas on the other hand, NF-κB p105 was simultaneously phosphorylated, releasing TPL2, leading to the activation of ERK1/2 through the phosphorylation of MEK1/2 (53). Notably, these two kinase cascades occur with identical activation profiles, leading to the phosphorylation of CREB reported to be downstream of p38 (14, 16, 63) and ERK1/2 (14, 15, 18). Significantly, a previous study using filarial Ag also linked CREB activation to the production of IL-10 (64). However, the full detail of these signaling pathways has not previously been reported in the context of helminth E/S molecules, and so for the first time, to our knowledge, we provide a complete molecular mechanism of IL-10 production by macrophages in response to schistosome E/S Ags.

The activation of the MAPK pathway described above links with the early detection of IL-10 mRNA in response to the 0–3hRP and matches the dynamics of 0–3hRP uptake by BMMs. In this study, we further demonstrate that the uptake of 0–3hRP is in part mediated by PI3K signaling. PI3K signaling is responsible for trafficking of proteins through different cellular compartments (65)—for example, the PI3K isoform p110-δ directs internalization of TLR4, which thereby limits this receptor’s ability to signal from the cell surface (66). PI3K signaling is also required for adequate recruitment of TIRAP to TLRs (67). Consequently, after PI3K inhibition in 0–3hRP–stimulated BMMs, the reductions in phosphorylation of ERK1/2, p38, and CREB, plus diminished IL-10 production, could be due to a combination of reduced 0–3hRP uptake, blocking TLR receptor trafficking, but also blocking adequate TIRAP–MyD88 recruitment. As such, PI3K signaling reveals itself as a crucial initial step in macrophage responses to cercarial E/S products and potentially to other TLR ligands.

Only a couple of studies provide direct evidence on the interaction between CREB and DNA from the IL-10 promoter, and these are based upon either a highly artificial system (18) or in conjunction with another transcription factor (16). In this study, it is demonstrated that in BMMs exposed to cercarial E/S products, p-CREB is recruited to the IL-10 promoter in a region that overlaps with the first exon of the gene, which differs from the findings of the latter study (16). The abundant levels of p-CREB and its strong interaction with the IL-10 promoter both place this transcription factor in a privileged place to effectively regulate the transcription of IL-10. Nevertheless, regulation exerted by CREB in BMMs exposed to 0–3hRP was not just limited to IL-10. At least three important transcription factors, *Egr1*, *Fos*, and *Nfkb2*, were also the target of CREB. Although *Egr1* regulates hundreds of other genes (68), including several cytokines (e.g., TNF-α, IL-2, and IL-12) (69), its role is not well defined in macrophages. In contrast, *Fos*, which is a component of the transcription factor AP-1 (70) implicated in various inflammatory settings (71), could be acting in concert with *Nfkb2* to induce the production of proinflammatory cytokines. Lastly, as the phosphorylation of NF-κB p65 was evident after exposure of macrophages to 0–3hRP, p65 could form a complex with p50 released as a consequence of p105 degradation and so form the p50/p65 heterodimer, a well-known transcription factor for cytokines such as IL-1β, IL-6, and, importantly, IL-12 (13, 72).

In addition to the link between 0–3hRP and IL-10 through TLR signaling, a large number of genes involved in “metabolic process” appear to be regulated by CREB, which targeted genes associated with components of glycolysis, the tricarboxylic acid cycle, and oxidative phosphorylation. Moreover, glucose uptake and hexokinase activity, but not lactate production, were increased in BMMs stimulated with 0–3hRP, implying that processing and responding to 0–3hRP is costly from an energy point of view and relays on oxidative phosphorylation and not anaerobic glycolysis. The interplay and intimate linkage between metabolism and the immune response is becoming increasingly apparent (73–78), with a particular role for TLR signaling in modulating metabolism (78, 79). Consequently, our findings add a mechanistic insight into the manner in which TLR ligands might impact the metabolism of macrophages. Therefore, transcription factors such as CREB, involved in cytokine expression, could also regulate key metabolic process in activated cells.

Finally, it is important to consider the in vitro observations reported above in the context of in vivo conditions following percutaneous infection of a mammalian host, although directly relating the two can be challenging (42). All *S. mansoni* life stages in the mammalian host produce immunomodulatory E/S products (80), and in this study, we show that E/S products released by invading *S. mansoni* larvae have a profound impact upon host macrophages. The role of tissue-resident macrophages versus blood-derived monocytes in infectious disease immune processes is contentious (81–84), yet in the skin, we show that tissue-resident F4/80^+^MHC-II^high^ macrophages, and not recruited monocytes, are the main source of IL-10 early in response to *S. mansoni* infection. IL-10 production by skin F4/80^+^MHC-II^high^ macrophages could be the result of several diverse signals (stimulation by pathogen-associated molecular patterns, release of host-derived danger signals resulting from tissue damage following parasite penetration of the skin, or wound-healing responses); however, our findings greatly support the role of cercarial E/S products in the induction of IL-10 production by tissue resident macrophages.

In summary, we present a detailed study of the molecular events that occur in BMMs following exposure to cercarial E/S products leading to the production of IL-10. This mechanism involves the activation of CREB downstream of TLR2 and TLR4 via the phosphorylation of p38 and ERK1/2. Additionally, we show that this mechanism is also responsible for the regulation of the metabolic state of macrophages, which provides an early in vitro demonstration of the role helminth Ags have in modulating metabolism downstream of TLRs. Finally, we show that the production of IL-10 by macrophages in response to *S. mansoni* cercarial E/S products extends beyond an in vitro phenomenon and is also evident in the skin tissue-resident macrophages, which rapidly produce IL-10 in vivo following exposure to invading schistosome larvae. This early and rapid release of IL-10 has the potential to greatly modulate the immune response in the skin by limiting inflammation and tissue damage, plus conditioning the microenvironment recruited immune cells will encounter as they infiltrate the infection site.

## Supplementary Material

Data Supplement
