# Supplementary material for: IL-10 Production in Macrophages Is Regulated by a TLR-Driven CREB-Mediated Mechanism That Is Linked to Genes Involved in Cell Metabolism
Source: J Immunol. 2015 Jun 26;195(3):1218–32. doi: 10.4049/jimmunol.1500146 (PMC4505959; doi:10.4049/jimmunol.1500146)
Supplement: Data Supplement [file JI_1500146.zip › JI_1500146_Supplemental_Figures_1.pdf]

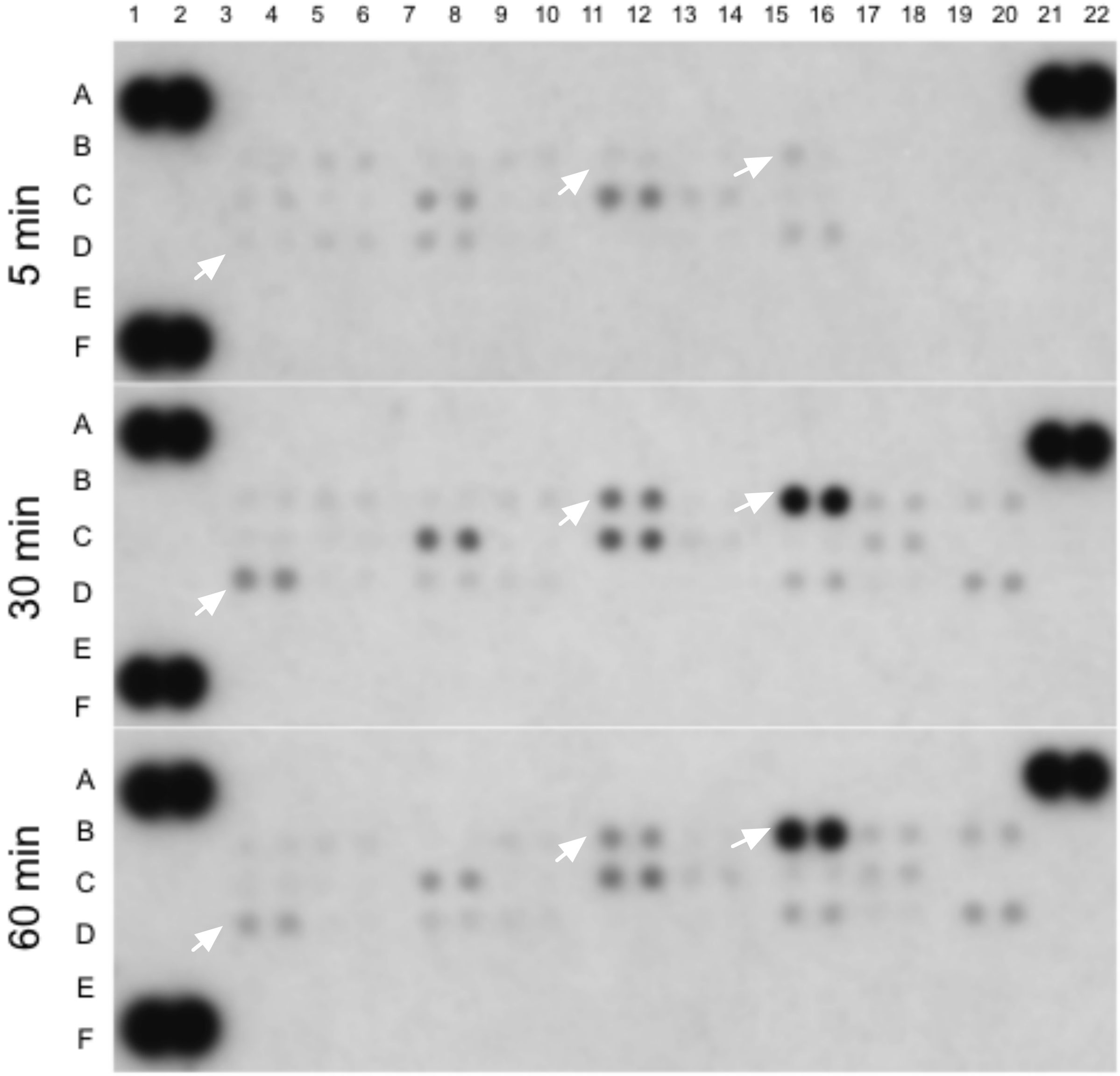

	1 2	3 4	5 6	7 8	9 10	11 12	13 14	15 16	17 18	19 20	21 22
A	(+)										(+)
B		Akt1	Akt2	Akt3	Akt pan	CREB	ERK1	ERK2	GSK-3α/β	GSK-3β	
C		HSP27	JNK1	JNK2	JNK3	JNK pan	MKK3	MKK6	MSK2		
D		p38α	p38β	p38δ	p38γ	p53	S6K1	RSK1	RSK2	TOR	
E										(-)	
F	(+)										

Figure S1. Kinase proteome profiler results from BMMs stimulated with cercarial E/S products

BMMs were stimulated with 50 µg/ml 0-3hRP supplemented with 2 µg/ml PMB for 5, 30 and 60 min. After stimulation, samples were prepared for analysis with the Proteome Profiler Array according to the manufacturer's instructions (R&D Systems, Abingdon, UK). Briefly, cells were lysed (30 min 4 °C), cell debris removed (800*g* 10 min) and final lysate mixed with biotinylated mAb cocktail and incubated for 1 hour. Lysate/mAb mixtures were then incubated with pre-blocked Proteome Profiler membranes overnight at 4 °C. The membranes were then washed with buffer and incubated with streptavidin conjugated to HRP. Chemiluminescence reagent revealed antibody labeling of proteins on the membranes using X-ray film imaging (GE Healthcare, Pittsburg, USA). Membranes from each time point and the coordinates for the proteins are presented; each protein is in duplicate in the array. Positive controls for the assay are in the corners.

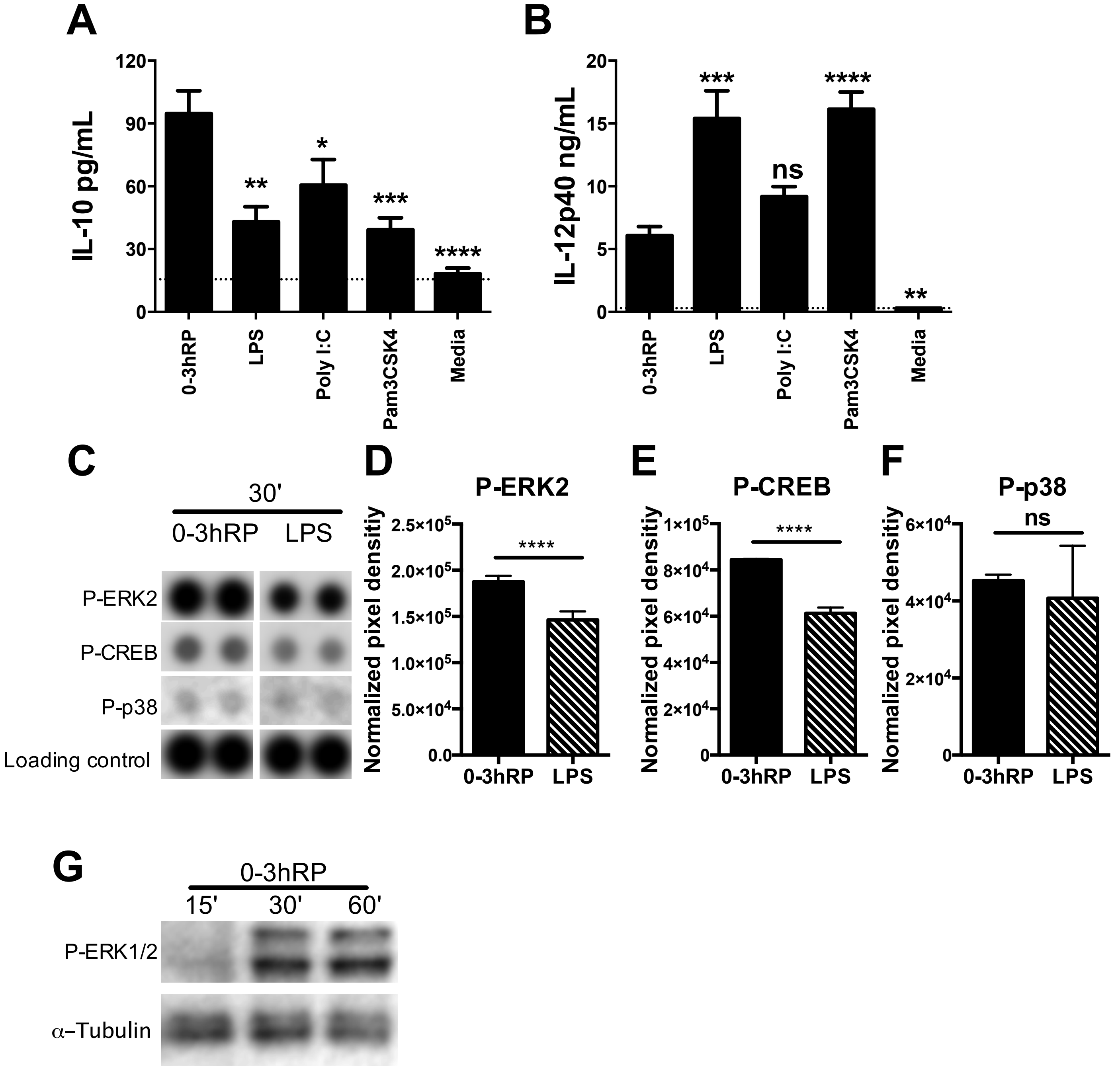

Figure S2. Comparison of BMMs stimulated with cercarial E/S products and other TLR ligands.

Protein levels of (**A**) IL-10 and (**B**) IL-12p40 in cultures of BMMs exposed to 50 µg/ml 0-3hRP, 1ng/ml LPS, 25 µg/ml Poly I:C, 5 µg/ml Pam3CSK4 or Media control. Bars represent mean from 3 biological replicates. ANOVA and Sidak's multiple comparisons test was performed to examine statistically significant differences between 0-3hRP treated BMMs and other TLR ligands (* = $p<0.05$; ** = $p<0.01$; *** = $p<0.001$; **** = $p<0.0001$; ns = $p>0.05$). Dotted lines represent lower detection limit of ELISA tests. ((**C**) Immunoblot (**D**-**F**) and densitometry analysis based on kinase proteome profiler array of phosphorylated (**D**) ERK2, (**E**) CREB and (**F**) p38 from BMMs stimulated for 30 minutes with 50 µg/ml 0-3hRP or 1ng/ml LPS. Bars represent normalized means based on loading controls from 4 biological replicates. Unpaired two tailed T-tests were performed to examine statistically significant differences between means of treated cells (**** = $p<0.0001$; ns = $p>0.05$).(**G**) Equivalent amounts of proteins from BMMs exposed to 50 µg/ml 0-3hRP for different lengths of time (15, 30 and 60 min) were analyzed by SDS-PAGE and western blotting with antibodies against phosphorylated ERK1/2 (P-ERK1/2) and α-Tubulin (as a loading control).

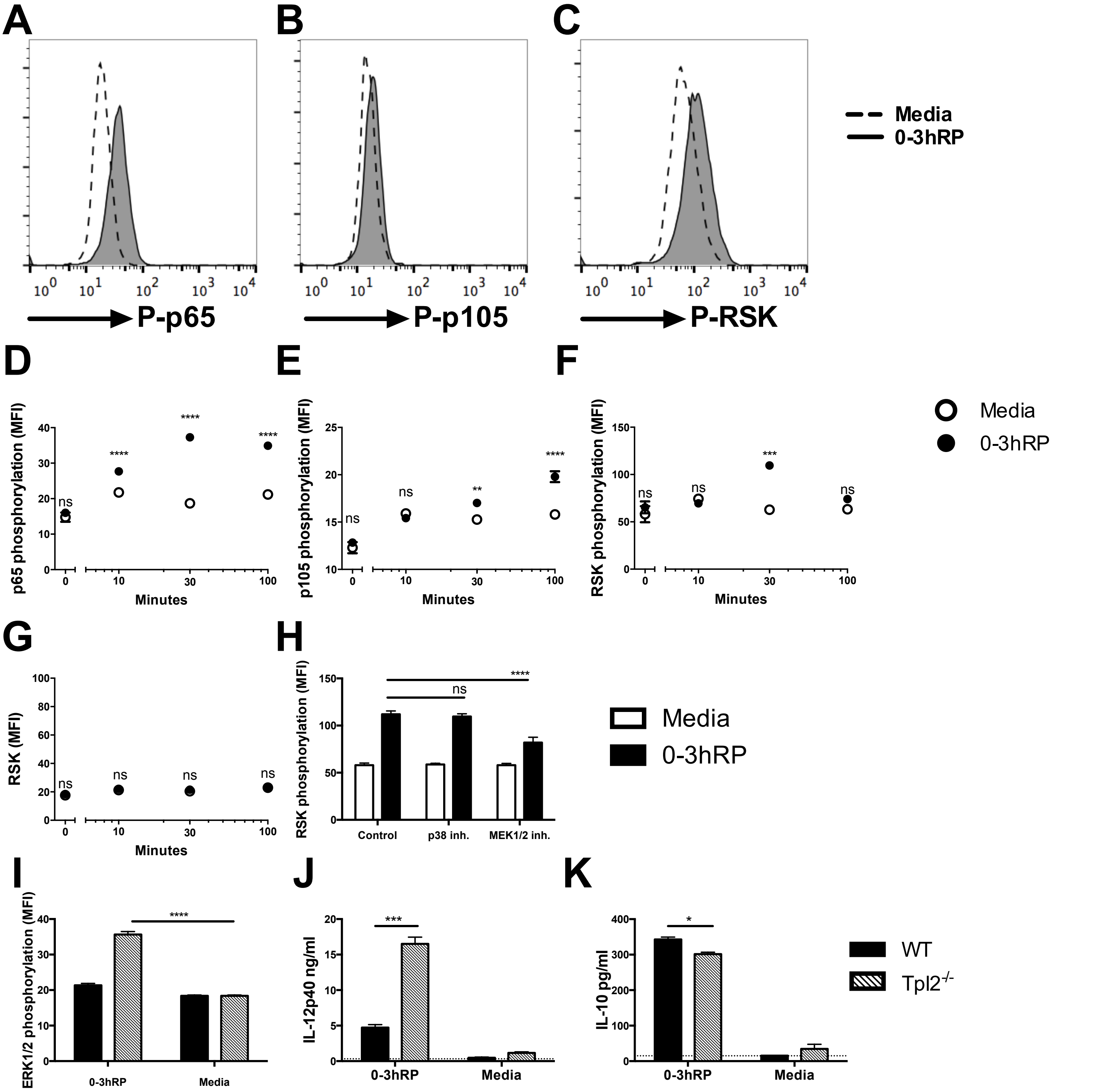

Figure S3. NF-κB (p65, p105) and TPL2/ERK/RSK activation in BMMs stimulated with 0-3hRP

(**A**-**C**) Representative overlaid flow cytometry histograms and (**D**-**I**) mean MFI ±SEM values for WT BMMs, or (**I**) Tpl2$^{-/-}$ BMMs, exposed to 50 µg/ml 0-3hRP (closed circles/bars) or Media control (open circles/bars) for (**A**-**C** & **H**-**I**) 30 min, or (**B** & **D**) 0-100 min. Cells were labeled with Abs against phosphorylated (**A** & **D**) p65, (**B** & **E**) p105, (**C**, **F** & **H**) phosphorylated RSK, (**G**) total RSK, or (**I**) phosphorylated ERK1/2. In selected experiments, (**H**) BMMs were treated with p38 inhibitor (SB203580, 1µM) or MEK inhibitor (U0126, 10µM) for 2 h before being exposed for 30 min to 50 µg/ml 0-3hRP. (**J**-**K**) Supernatants from WT (closed bars) or Tpl2$^{-/-}$ (hatched bars) BMM cultures exposed overnight to 50 µg/ml 0-3hRP or Media control were tested for the presence of (**J**) IL-12p40 and (**K**) IL-10. Symbols and bars are means of 3 biological replicates. Results are representative of three independent experiments. ANOVA and (Sidak's and Tukey's) were performed to examine statistically significant differences (**D**-**G**) between 0-3hRP treated BMMs and corresponding Media control at each time point, or (**H**-**K**) between the means of selected groups (* = $p<0.05$; ** = $p<0.01$; *** = $p<0.001$; **** = $p<0.0001$; ns = $p>0.05$). Dotted line represent detection limit in ELISA tests.
